# Leaf Traits, Yield Components, and Seed Quality of *Paeonia ostii* Across Contrasting Understory Positions in a Pecan-Based Agroforestry System

**DOI:** 10.3390/plants15182875

**Published:** 2026-09-20

**Authors:** Jiaojiao Zhang, Donglin Li, Yu Duan, Haile Niu, Ru Li

**Affiliations:** 1 Jiangsu Academy of Forestry, Nanjing 211153, China; white@warmbook.net (J.Z.); kyc@jaf.ac.cn (D.L.); 2 College of Forestry and Grass, Nanjing Forestry University, Nanjing 210037, China; 3 College of Life Sciences and Food Engineering, Huai’an University, Huai’an 223003, China

**Keywords:** *Paeonia ostii*, *Carya illinoinensis*, understory light intensity, leaf acclimation, yield formation, seed quality

## Abstract

To evaluate trait variation across contrasting understory light environments in a pecan-based agroforestry system, 30 seven-year-old *Paeonia ostii* plants were assessed at three naturally occurring positions with high (HI), medium (MI), and low (LI) understory light intensity. Microenvironmental conditions, plant growth, leaf photosynthetic pigments and mineral nutrients, seed yield components, seed oil traits, and final germination percentage were measured. Photosynthetically active radiation consistently followed the order HI > MI > LI. Plants at LI had higher chlorophyll a, chlorophyll b, carotenoid, and leaf N, P, and K concentrations, indicating leaf-level responses to lower light availability. In contrast, plant height and crown width were lower at LI, while HI showed higher values for 100-seed weight, aggregate fruit number and fresh weight, seeds per plant, and final germination percentage. Ground diameter and seed diameter did not differ significantly among positions. Crude fat and α-linolenic acid contents also showed no significant differences. Integrated trait analysis further showed that HI was associated mainly with higher growth, reproductive, and germination traits, whereas LI was associated with higher leaf pigment and mineral nutrient concentrations. Overall, the results reveal contrasting leaf-level acclimation and whole-plant performance across a naturally occurring understory PAR gradient. Because the study was observational, these patterns should be interpreted as position-associated differences rather than causal effects of light, and they provide a basis for future replicated canopy-management experiments.

## 1. Introduction

*Paeonia ostii* is a perennial woody oilseed species with a long cultivation history and broad environmental suitability in China [1,2]. Unlike annual oil crops such as soybean (*Glycine max*), rapeseed (*Brassica rapa*), and peanut (*Arachis hypogaea*), *P. ostii* can provide repeated harvests after establishment. Considerable variation in agronomic and seed oil traits among tree peony cultivars also indicates substantial potential for crop improvement and utilization [3]. Its seed oil is rich in unsaturated fatty acids, particularly α-linolenic acid, which is an important determinant of its nutritional value and development potential [4,5,6]. Previous studies have also described seed development and the accumulation of storage reserves in *P. ostii* [7]. However, the production performance of this species depends not only on its genetic characteristics but also on environmental conditions during vegetative growth, fruit development, and seed filling.

Previous studies on *P. ostii* cultivation have mainly examined nutrient management and reproductive development. Leaf nutrient diagnosis has been used to guide fertilization during key developmental stages, including fruit expansion [8], while the timing and balance of N, P, and K supply can influence phenological development, biomass accumulation, and plant growth [9]. Nitrogen application may alter source–sink nitrogen metabolism and seed oil quality, whereas phosphorus availability can affect photosynthetic performance, fatty acid composition, and seed yield [10,11]. Changes in starch and sucrose metabolism during seed development have been associated with variation in seed yield in *P. ostii* [12]. In addition, pollination success affects fruit set, fruit growth, and final seed yield [13,14]. Together, these findings indicate that yield and seed quality in *P. ostii* depend on the coordination of nutrient acquisition, assimilate supply, fruit development, and seed filling. However, most previous studies have examined these processes separately or under relatively uniform cultivation conditions, and their responses to the spatially heterogeneous environments of agroforestry systems remain poorly understood.

Light strongly influences plant carbon assimilation, morphology, and reproductive development. In addition to supplying energy for photosynthesis, light regulates chlorophyll synthesis, carbohydrate metabolism, and biomass allocation [15]. Under field conditions, understory irradiance varies with canopy structure, solar elevation, and temporal fluctuations in incident light [16]. Plants exposed to limited light often adjust chlorophyll concentration and light-harvesting capacity, which may enhance light capture under shaded conditions [17]. These adjustments help leaves acclimate to low light, but they may not offset the reduction in whole-plant carbon gain. In *P. ostii*, shading has been reported to alter photosynthetic characteristics, growth, and seed production. Mild shading may benefit some physiological and seed-quality traits, whereas moderate or severe shading can reduce fruit production, seed filling, and nutrient accumulation [18,19]. Responses to understory light conditions therefore need to be evaluated not only at the leaf level but also in terms of plant growth, reproductive output, seed composition, and germination performance.

*P. ostii* is increasingly being introduced into orchard-based agroforestry systems. These systems can improve land-use efficiency and may moderate high temperatures and reduce water loss during summer. At the same time, the overstory redistributes solar radiation, creating marked differences in light availability among understory positions. Tree-based agroforestry has been shown to influence photosynthesis, growth, and yield in *P. ostii* [20], while intercropping with pecan (*Carya illinoinensis*) may alleviate heat stress during periods of high temperature [21]. More broadly, tree–crop interactions can alter the light environment, physiological performance, and productivity of understory crops [22]. Therefore, the suitability of an understory position depends on whether the local light environment allows plants to acclimate without compromising reproductive performance.

Despite growing interest in *P. ostii* cultivation in agroforestry systems, its responses to contrasting understory light-intensity conditions beneath pecan canopies have not been comprehensively evaluated. In particular, it remains unclear how variation in understory light intensity and associated microclimatic conditions is related to leaf traits, vegetative growth, aggregate fruit and seed development, seed oil quality, and final germination percentage. In this study, seven-year-old *P. ostii* plants in an established pecan-based agroforestry system were compared among three representative understory positions differing in light intensity: high light intensity (HI), medium light intensity (MI), and low light intensity (LI). Photosynthetically active radiation, air temperature, relative humidity, plant growth traits, leaf photosynthetic pigment and mineral nutrient contents, seed yield components, crude fat content, selected fatty acids, and final germination percentage were measured. Standardized heatmap and correlation analyses were used to assess integrated trait patterns and trait covariation. This study aimed to clarify the relationships among understory light intensity, associated microclimatic conditions, leaf traits, plant growth, reproductive performance, and seed quality, and to provide a basis for future replicated canopy-management studies.

## 2. Materials and Methods

### 2.1. Experimental Site

The experiment was conducted at Zhonghua Peony Garden, Weiwei Village, Wangji Town, Siyang County, Suqian City, Jiangsu Province, China (33°51′54″ N, 118°48′05″ E). The region is located in the transitional zone of the northern subtropical monsoon climate. According to meteorological records from the Siyang National-level Meteorological Observation Station for 2014–2024, the mean annual temperature, annual precipitation, and annual sunshine duration were 14.2 °C, 917.8 mm, and 2168.3 h, respectively. The soil at the experimental site was a slightly acidic sandy red soil.

In 2018, Jiangsu Peony Technology Co., Ltd. conducted uniform site preparation by adding soil and increasing soil depth to reduce spatial variation in soil conditions across the experimental area. In the same year, 30 grafted pecan trees of the cultivar ‘Pawnee’ (*Carya illinoinensis* ‘Pawnee’) were transplanted at a spacing of 8 m × 8 m. At the time of the study, the pecan trees had a mean total height of approximately 5.0 m and a mean crown width of approximately 7.5 m. In 2019, a total of 150 one-year-old *P. ostii* ‘Feng Dan’ seedlings, raised from seeds in nursery beds, were transplanted into the experimental field and beneath the pecan canopy at a spacing of 1.0 m × 0.8 m.

Pecan tree dimensions were measured on 10 July 2025 for 30 trees. Tree height was measured using a tree height meter. Crown width was measured along two perpendicular directions (east–west and north–south) using a measuring tape, and the mean of the two measurements was used as the crown width of each tree. The reported orchard-level values represent the means of the measured trees.

### 2.2. Plant Materials and Classification of Understory Light Intensity

Seven-year-old *P. ostii* ‘Feng Dan’ plants from the 2019 planting batch were used in this study. Spatial variation in pecan canopy openness created naturally contrasting levels of understory light intensity. Based on preliminary field observations and measurements of photosynthetically active radiation (PAR), three representative understory positions were classified as high light intensity (HI), medium light intensity (MI), and low light intensity (LI). Hereafter, HI, MI, and LI denote the sampled understory positions with high, medium, and low light intensity, respectively, rather than experimentally imposed light treatments. Because the three light-intensity categories represented naturally occurring spatial positions within a single orchard rather than independently replicated light treatments, the study was observational in design. The sampled plants within each position were therefore treated as plant-level observations rather than independent replicates of a light treatment.

Plants at the HI position were located in relatively open spaces between pecan crowns and received little canopy shade. Plants at the MI position were located near the canopy edge and received partial shade. Plants at the LI position were located beneath denser canopy cover and had the lowest measured PAR.

Ten healthy *P. ostii* plants without visible symptoms of pests or diseases were selected from each light-intensity position, giving 30 sampled plants in total. All selected plants were of the same age, planted at the same spacing, and managed using the same cultivation practices.

### 2.3. Measurement of Microenvironmental Variables

Three fixed monitoring points were set within each of the HI, MI, and LI positions, giving nine monitoring points in total. All measurements were taken 1.0 m above the ground, approximately at the canopy height of the sampled *P. ostii* plants.

Microenvironmental measurements were conducted on 14 July and 10 November 2025 under clear skies and calm or light-wind conditions. At each monitoring point, photosynthetically active radiation (PAR), air temperature, and relative humidity were recorded at 2 h intervals from 07:00 to 19:00 using LI-190R quantum sensors and HMP155A temperature–humidity probes (LI-COR Biosciences, Lincoln, NE, USA; Vaisala, Vantaa, Finland).

### 2.4. Measurement of Growth Traits

Growth traits were measured on 25 June 2025 using the 10 labeled *P. ostii* plants at each of the three light-intensity positions. Plant height was measured from the soil surface to the highest point of the canopy using a measuring tape. Ground diameter was measured at the stem base using a digital vernier caliper. Crown width was measured in the east–west and north–south directions, and the mean of the two measurements was recorded for each plant.

### 2.5. Measurement of Leaf Photosynthetic Pigments and Mineral Nutrient Concentrations

Leaf samples were collected on 25 June 2025 from the 10 labeled plants at each light-intensity position. Fully expanded, healthy leaves of similar maturity were collected from the middle portion of the outer canopy. Leaves from the east, west, south, and north sides of each plant were combined as one biological replicate, giving 10 sampled plants per light-intensity position.

Each sample was divided into two portions. Fresh leaves were used immediately for pigment analysis, while the remaining material was dried at 80 °C to constant weight, ground to a fine powder, and used for mineral nutrient analysis.

The contents of chlorophyll a, chlorophyll b, and carotenoids were determined according to the method of Lichtenthaler and Wellburn [23]. Fresh leaf tissue was extracted with 80% acetone, and absorbance was measured using a UV–Vis spectrophotometer (UV-1800, Shimadzu Corporation, Kyoto, Japan). The measured leaf mineral nutrient traits were total N, total P, and total K. After digestion with concentrated sulfuric acid and hydrogen peroxide, total nitrogen content was determined using the Kjeldahl method, total phosphorus content was determined using an automated discrete chemical analyzer (Smartchem 200, WestCo Scientific Instruments, Brookfield, CT, USA), and total potassium content was determined using the flame photometry method [24]. Photosynthetic pigment concentrations were expressed on a fresh-mass basis (mg/g FW), whereas leaf N, P, and K concentrations were expressed on a dry-mass basis (g/kg DW). The chlorophyll a/b ratio was calculated for each individual plant before averaging within each position.

### 2.6. Measurement of Seed Yield Components

Peak bloom occurred from 14 to 20 April at HI, from 18 to 23 April at MI, and from 22 to 28 April at LI, with flowering at LI occurring approximately 7–10 days later than at HI. Because plants from all three positions were harvested on the same calendar date in late July, the developmental duration from peak bloom to harvest also differed among positions, corresponding approximately to 100–105 days at HI, 96–101 days at MI, and 92–97 days at LI. Thus, seeds collected from the more heavily shaded positions may have been at an earlier developmental stage at harvest.

Mature aggregate fruits were harvested in late July 2025 from the 10 labeled *P. ostii* plants at each light-intensity position. Aggregate fruits and seeds from different plants were kept separately. The measured traits included aggregate fruit per plant, aggregate fruit fresh weight, aggregate fruit maximum transverse diameter, seeds per plant, 100-seed weight, and seed diameter.

All mature aggregate fruits produced by each plant were harvested and counted. After the seeds were removed, the number of mature seeds from each plant was recorded. The 100-seed weight in the present study was determined using fresh seeds randomly sampled from each individual plant immediately after fruit harvest. The fresh weight of individual aggregate fruits and 100-seed weight were measured using an electronic balance (Kubei Co., Ltd., Shanghai, China; capacity: 510 g, readability: 0.001 g), whereas the maximum transverse diameter of individual aggregate fruits and seed diameter were measured using a digital vernier caliper (Guilin Measuring & Cutting Tool Co., Ltd., Guilin, China; readability: 0.01 mm). The mean values were calculated separately for each plant.

### 2.7. Measurement of Crude Fat Content and Fatty Acid Composition

Mature seeds from each labeled plant were kept separately, air-dried, and cleaned of impurities and visibly unfilled seeds. Crude fat content was determined using a minispec mq20 low-field time-domain nuclear magnetic resonance analyzer (Bruker BioSpin, Ettlingen, Germany; 20 MHz, 0.47 T) with whole seeds (intact seeds including the seed coat and kernel) that had been oven-dried to a moisture content below 10%. Depending on seed size, a 4–8 g sample of dried whole seeds was weighed to the nearest 0.001 g and analyzed using an instrument calibration curve established with seed samples known for their oil content. Crude fat was calculated on a whole-seed dry-weight (DW) basis and expressed as % (g per 100 g dry weight), with the dry mass of whole seeds serving as the calculation denominator. Fatty acids were extracted from dissected de-hulled seed kernels, quantified following the national food safety standard GB 5009.168—2016 (Determination of Fatty Acids in Foods), and identified and quantified via the internal-standard method. Analyses were calculated on a kernel dry-weight (DW) basis and reported in units of g/kg or mg/g, with the dry mass of seed kernels serving as the calculation denominator. The content of α-linolenic acid was further determined among the detected fatty acid components.

### 2.8. Measurement of Final Germination Percentage

Mature yellow-brown aggregate fruits were collected from the labeled plants, air-dried indoors, and allowed to after-ripen. Fifty healthy, fully developed seeds from each plant were placed together in one germination box containing sterilized sand. The sand was maintained in a moist condition throughout stratification by periodically adding distilled water as needed. Stratification was initiated on 4 August 2025 at 15 °C to promote radicle emergence. Radicle emergence was recorded from day 30 at 7-day intervals, and the final assessment was conducted on day 98 (10 November 2025). The germinated seeds were then transferred to 4 °C until epicotyl emergence and subsequently moved to greenhouse conditions for further growth. Germination percentage (GP, %) was calculated according to Equation (1):

$$\mathrm{GP}\;=\;\frac{N_{g}}{N_{t}}\;\times \;100$$

where N_g_ is the number of germinated seeds and N_t_ is the total number of tested seeds.

### 2.9. Data Processing and Statistical Analysis

Data were organized in Excel 2016 and analyzed using R version 4.5.3. Because HI, MI, and LI represented naturally occurring positions within a single orchard rather than independently replicated light treatments, statistical comparisons were interpreted as descriptive differences among sampled positions. For continuous traits, normality and homogeneity of variance were assessed using the Shapiro–Wilk and Levene’s tests, respectively. Data meeting both assumptions were analyzed by one-way ANOVA followed, when significant, by Fisher’s protected LSD test. Welch’s ANOVA with Games–Howell comparisons was used for heterogeneous variances, whereas the Kruskal–Wallis test followed by Dunn’s test with Benjamini–Hochberg adjustment was used when normality was not met. Final germination was analyzed using a binomial GLM based on the numbers of germinated and non-germinated seeds (50 seeds per plant). Pearson correlations were calculated both across all 30 plants and after removing position effects, with Benjamini–Hochberg FDR correction applied to correlation *p* values. Microenvironmental data were summarized descriptively as mean ± SD without inferential testing. An additional binomial GLM was used to examine the effects of position and 100-seed weight upon final germination, with their interaction tested before fitting the additive model. Z-score standardization was used for the integrated heatmap analysis. Statistical analyses were conducted using the R packages car, agricolae, rstatix, emmeans, and ggplot2, together with base R functions.

## 3. Results

### 3.1. Differences in Microenvironmental Variables Among Understory Positions Differing in Light Intensity

Measurements were conducted on 10 November (late autumn) in the northern subtropical monsoon zone. At this time point, pecan trees still maintained green foliage, whereas *P. ostii* ‘Feng Dan’ had shed leaves and entered dormancy. Although seed harvest of Feng Dan was completed in late July, this measurement aimed to capture late-season microclimate variations under pecan canopy within the intercropping system.

**Figure 1:**
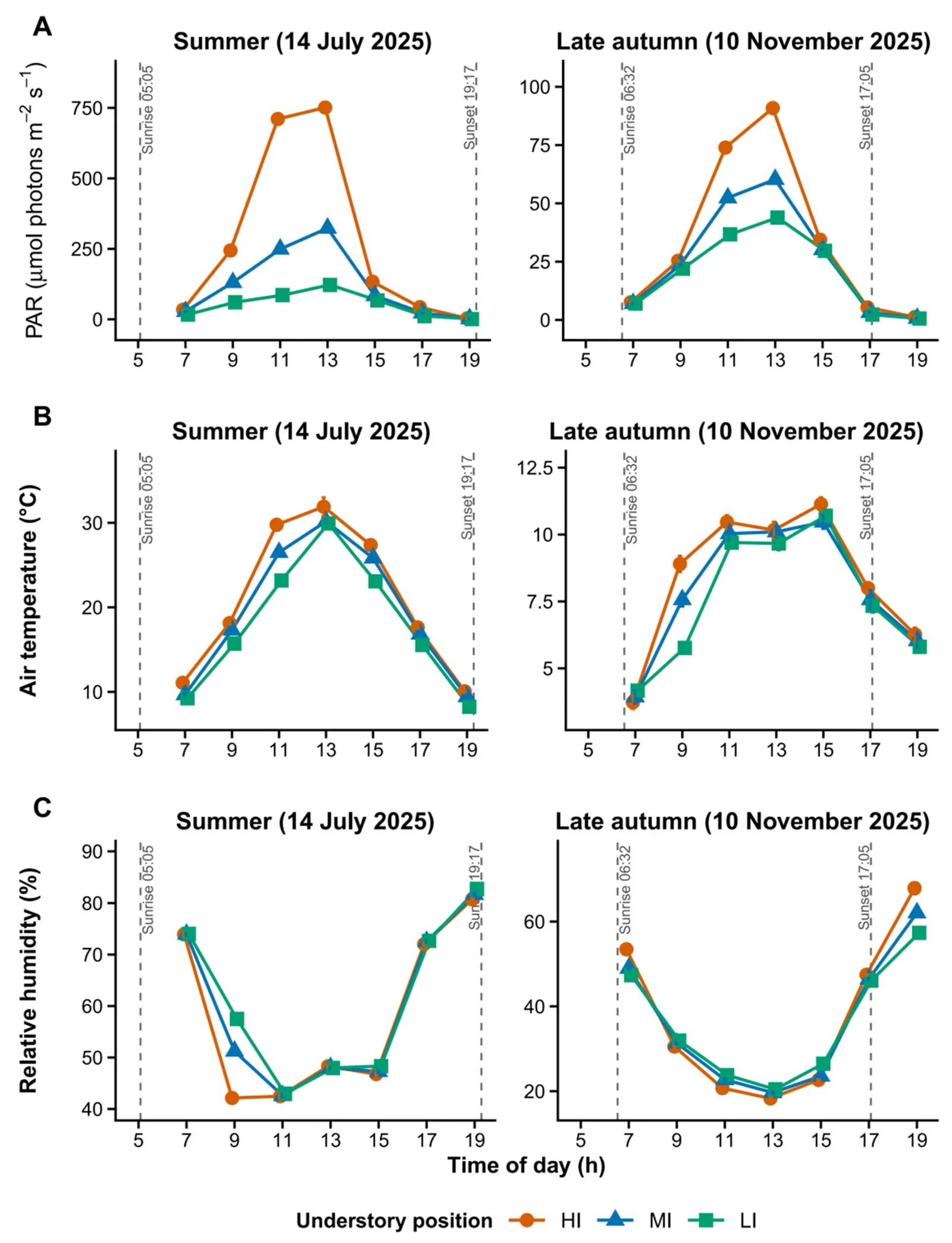
Diurnal variation in microenvironmental variables at three understory positions differing in light intensity during summer and late autumn. (**A**) Photosynthetically active radiation; (**B**) air temperature; (**C**) relative humidity. Values are means ± SD (*n* = 3 monitoring points per position). Measurements were obtained using LI-190R quantum sensors and HMP155A temperature–humidity probes. Dashed lines indicate local civil sunrise and sunset (China Standard Time, UTC+8). HI, MI, and LI indicate positions with high-, medium-, and low-understory light intensity, respectively.

Photosynthetically active radiation showed a unimodal diurnal pattern in both summer and late autumn, peaking at 13:00 (Figure 1A). In summer, PAR consistently followed the order HI > MI > LI at all measurement times. At 13:00, PAR reached 751.17, 323.30, and 122.37 μmol photons m^−2^ s^−1^ at HI, MI, and LI, respectively. At the same time, open-sky PAR was 1865 μmol photons m^−2^ s^−1^, corresponding to 40.3%, 17.3%, and 6.6% of full-sun PAR at HI, MI, and LI, respectively. The corresponding mean daytime PAR values were 274.31, 120.04, and 52.35 μmol photons m^−2^ s^−1^.

Late-autumn PAR was substantially lower than summer PAR, while the order HI > MI > LI remained unchanged. At 13:00, PAR was 90.87, 60.27, and 43.90 μmol photons m^−2^ s^−1^ at HI, MI, and LI, respectively. The corresponding mean daytime PAR values were 34.11, 25.35, and 20.30 μmol photons m^−2^ s^−1^. Relative to HI, mean daytime PAR at MI and LI was 43.8% and 19.1%, respectively, in summer, and 74.3% and 59.5%, respectively, in late autumn. Together, these measurements confirmed a clear gradient in PAR among the three understory positions.

Air temperature increased from morning to early afternoon and then declined (Figure 1B). In summer, the temperature at HI reached 31.90 °C at 13:00, compared with lower values at MI and LI. Mean daily temperatures were 20.85, 19.39, and 17.84 °C at HI, MI, and LI, respectively. Late autumn temperatures were lower and showed less variation among the three positions. At 09:00, temperatures were 8.90, 7.57, and 5.77 °C at HI, MI, and LI, respectively. At 11:00, temperature was highest at HI and lowest at LI, with MI showing an intermediate value. Mean daily temperatures in late autumn were 8.38, 7.96, and 7.59 °C at HI, MI, and LI, respectively.

Relative humidity generally varied inversely with PAR and air temperature, decreasing toward midday and increasing again in the late afternoon (Figure 1C). In summer, relative humidity was similar among the three positions at 07:00, 11:00, and 13:00. At 09:00 and 19:00, relative humidity followed the order HI < MI < LI. At 15:00, relative humidity was higher at LI than at HI and MI, whereas at 17:00, LI was higher than HI and MI was intermediate. Mean daily relative humidity was 58.05%, 59.61%, and 60.88% at HI, MI, and LI, respectively.

In late autumn, relative humidity followed the order HI > MI > LI at 07:00 and 19:00, whereas the order HI < MI < LI was observed from 09:00 to 15:00. At 17:00, relative humidity was higher at HI than at MI and LI, whereas MI and LI were similar. Mean daily relative humidity was 37.29%, 36.40%, and 36.21% at HI, MI, and LI, respectively. Overall, PAR showed the clearest separation among the three light-intensity positions, whereas differences in air temperature and relative humidity were smaller and varied with time of day.

### 3.2. Growth Traits Across Understory Positions Differing in Light Intensity

**Figure 2:**
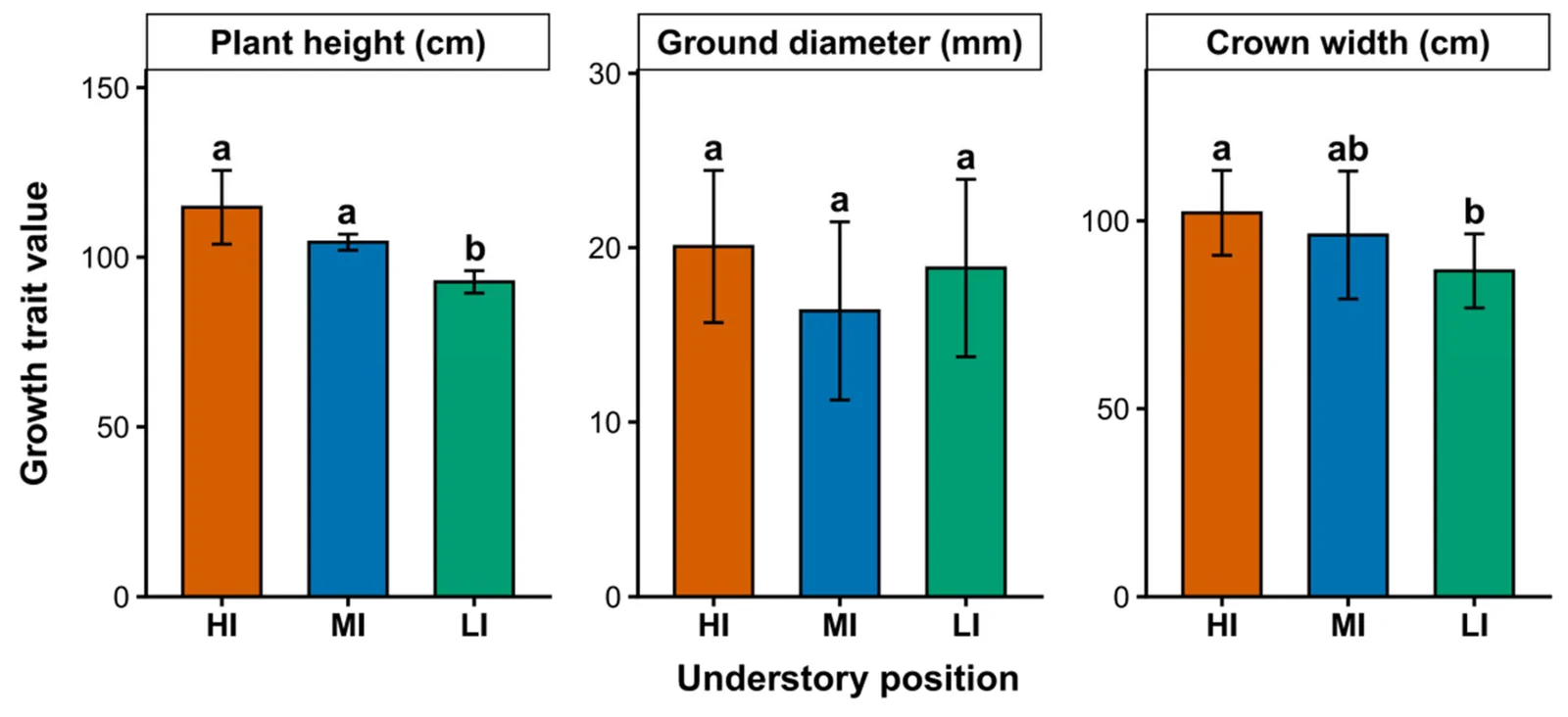
Growth traits of *P. ostii* at three understory positions differing in light intensity. Values are means ± SD (*n* = 10). Different lowercase letters indicate significant differences among positions at *p* < 0.05.

Plant height showed a decreasing pattern from HI to LI, with mean values of 114.70, 104.40, and 92.70 cm at HI, MI, and LI, respectively. Plant height was significantly lower at LI than at HI and MI, whereas HI and MI did not differ significantly (overall *P* = 1.58 × 10^−5^; Figure 2). Ground diameter showed a non-monotonic pattern, with mean values of 20.06, 16.37, and 18.83 mm at HI, MI, and LI, respectively, corresponding to the order HI > LI > MI; however, the differences among the sampled positions were not significant (*p* > 0.05). Crown width was 102.11, 96.23, and 86.65 cm at HI, MI, and LI, respectively. Crown width was significantly greater at HI than at LI, whereas MI was intermediate and did not differ significantly from either position. Overall, plant height and crown width were higher at HI than at LI, whereas ground diameter showed no significant difference and did not follow the PAR gradient among the three sampled positions.

### 3.3. Leaf Photosynthetic Pigments and Mineral Nutrient Concentrations Across Understory Positions Differing in Light Intensity

**Figure 3:**
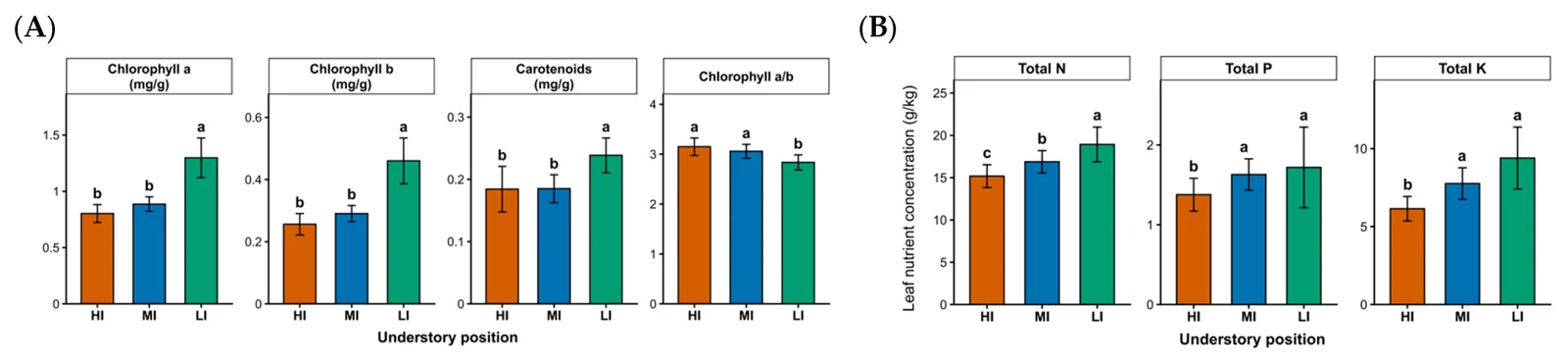
Leaf photosynthetic pigment and mineral nutrient concentrations of *P. ostii* at three understory positions differing in light intensity. (**A**) Photosynthetic pigment traits; (**B**) leaf N, P, and K concentrations. Values are means ± SD (*n* = 10). Different lowercase letters indicate significant differences at *p* < 0.05.

Leaf photosynthetic pigment concentrations increased as understory light intensity decreased (Figure 3A). The concentrations of chlorophyll a, chlorophyll b, and carotenoids were significantly higher at LI than at HI and MI, whereas HI and MI were similar. The corresponding values at HI, MI, and LI were 0.80, 0.89, and 1.30 mg/g for chlorophyll a; 0.26, 0.29, and 0.46 mg/g for chlorophyll b; and 0.18, 0.19, and 0.24 mg/g for carotenoids, respectively. The chlorophyll a/b ratio was 3.15, 3.06, and 2.83 at HI, MI, and LI, respectively. The ratio was significantly lower at LI than at HI and MI, whereas HI and MI did not differ significantly (*P* = 3.55 × 10^−4^).

Leaf N, P, and K concentrations generally increased as understory light intensity decreased, although differences in total P were less pronounced than those in total N and total K (Figure 3B). Total N increased significantly from 15.18 g/kg at HI to 16.88 g/kg at MI and 18.93 g/kg at LI (*P* = 8.09 × 10^−5^). Total K concentrations were 6.14, 7.76, and 9.40 g/kg at HI, MI, and LI, respectively. Total K was significantly lower at HI than at MI and LI, whereas MI and LI did not differ significantly (*P* = 1.66 × 10^−4^). Total P concentrations were 1.38, 1.63, and 1.72 g/kg at HI, MI, and LI, respectively. Total P was significantly lower at HI than at MI and LI, whereas MI and LI did not differ significantly (*p* = 0.038).

### 3.4. Seed Yield Components Across Understory Positions Differing in Light Intensity

**Figure 4:**
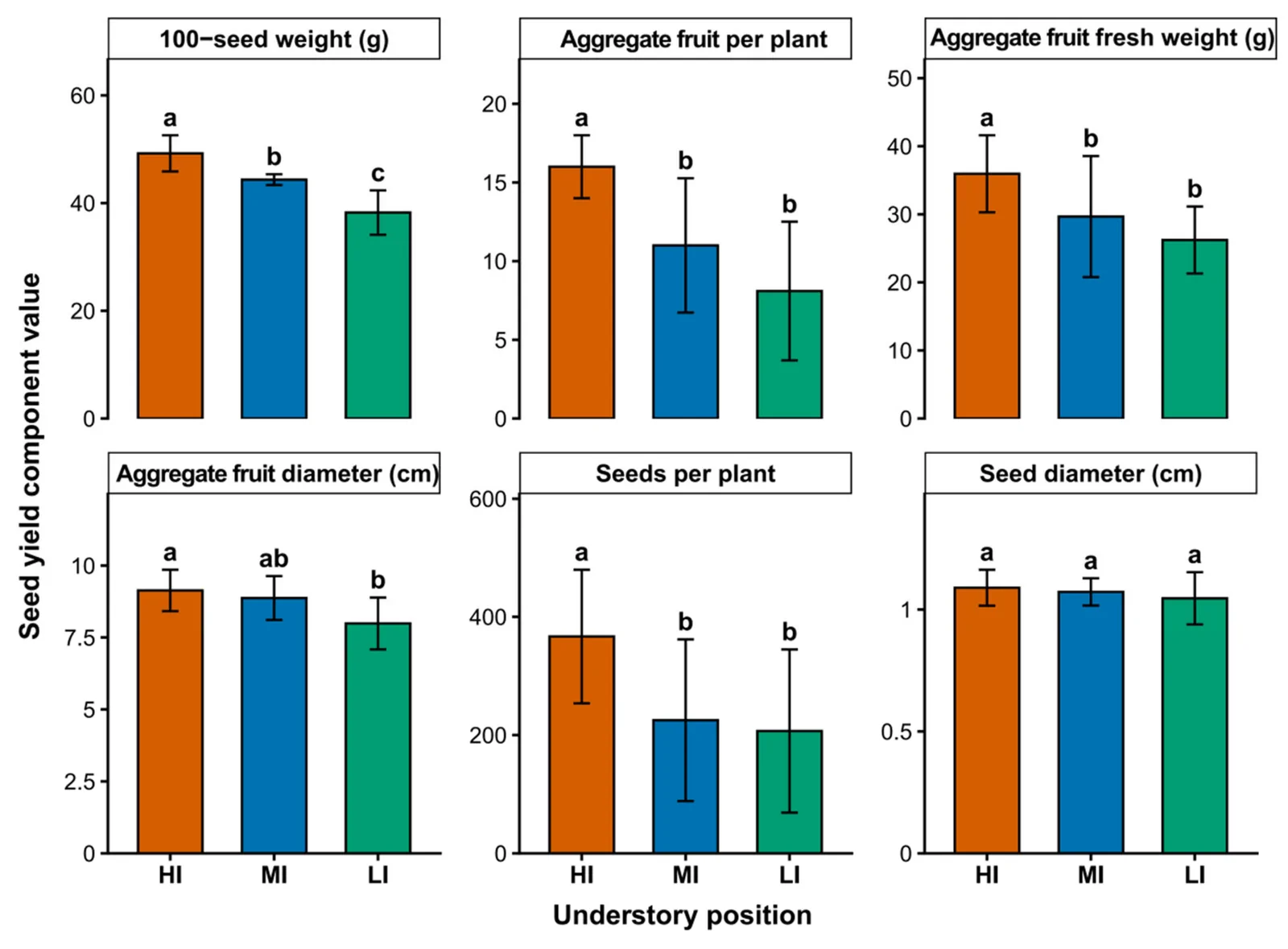
Seed yield components of *P. ostii* at three understory positions differing in light intensity. Values are means ± SD (*n* = 10). Different lowercase letters indicate significant differences among positions at *p* < 0.05.

Most seed yield components generally declined as understory light intensity decreased, although the magnitude and statistical significance of the differences varied among traits (Figure 4). The 100-seed weight was 49.23, 44.35, and 38.24 g at HI, MI, and LI, respectively, with significant differences among the three positions (*P =* 9.85 × 10^−8^).

Plants at HI produced significantly more aggregate fruit per plant (16.03) than those at MI (11.02) and LI (8.17), whereas MI and LI were similar. Aggregate fruit fresh weight showed the same pattern, with values of 35.95, 29.66, and 26.22 g at HI, MI, and LI, respectively. Aggregate fruit maximum transverse diameter was 9.14, 8.87, and 7.99 cm at HI, MI, and LI, respectively. It was significantly greater at HI than at LI, whereas MI was intermediate and did not differ significantly from either position (*P* = 0.020).

Plants at HI also produced significantly more seeds per plant (366.75) than those at MI (225.13) and LI (206.86), whereas MI and LI were similar. Seed diameter was similar among the three positions, with values of 1.09, 1.07, and 1.05 cm at HI, MI, and LI, respectively.

### 3.5. Seed Oil Quality and Final Germination Percentage Across Understory Positions Differing in Light Intensity

**Figure 5:**
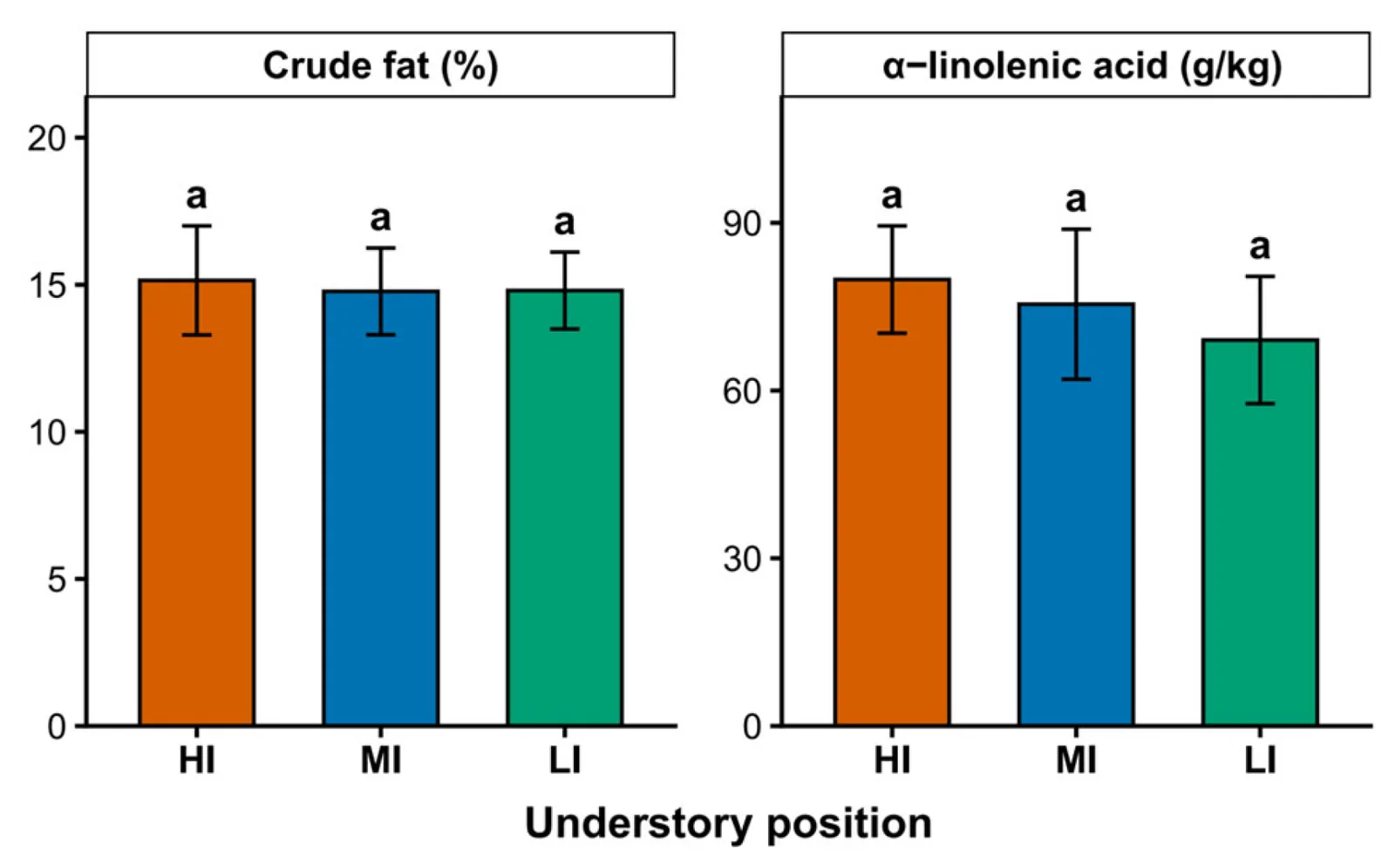
Crude fat and α-linolenic acid contents of seeds from *P. ostii* plants at three understory positions differing in light intensity. Values are means ± SD (*n* = 10). Different lowercase letters indicate significant differences among positions at *p* < 0.05.

Crude fat content did not differ significantly among the three positions (*P* = 0.839; Figure 5), with values of 15.15%, 14.77%, and 14.80% at HI, MI, and LI, respectively. α-Linolenic acid content was 79.85, 75.44, and 69.04 g/kg at HI, MI, and LI, respectively, but the differences among positions were not significant (*P* = 0.096). It should be noted that crude fat was quantified on a whole-seed basis, including the lipid-poor seed coat, whereas fatty acid profiles were determined using dissected seed kernels. Most published tree-peony seed-oil data are reported for decorticated kernels, which partly accounts for the lower absolute crude fat values observed in the present study.

**Figure 6:**
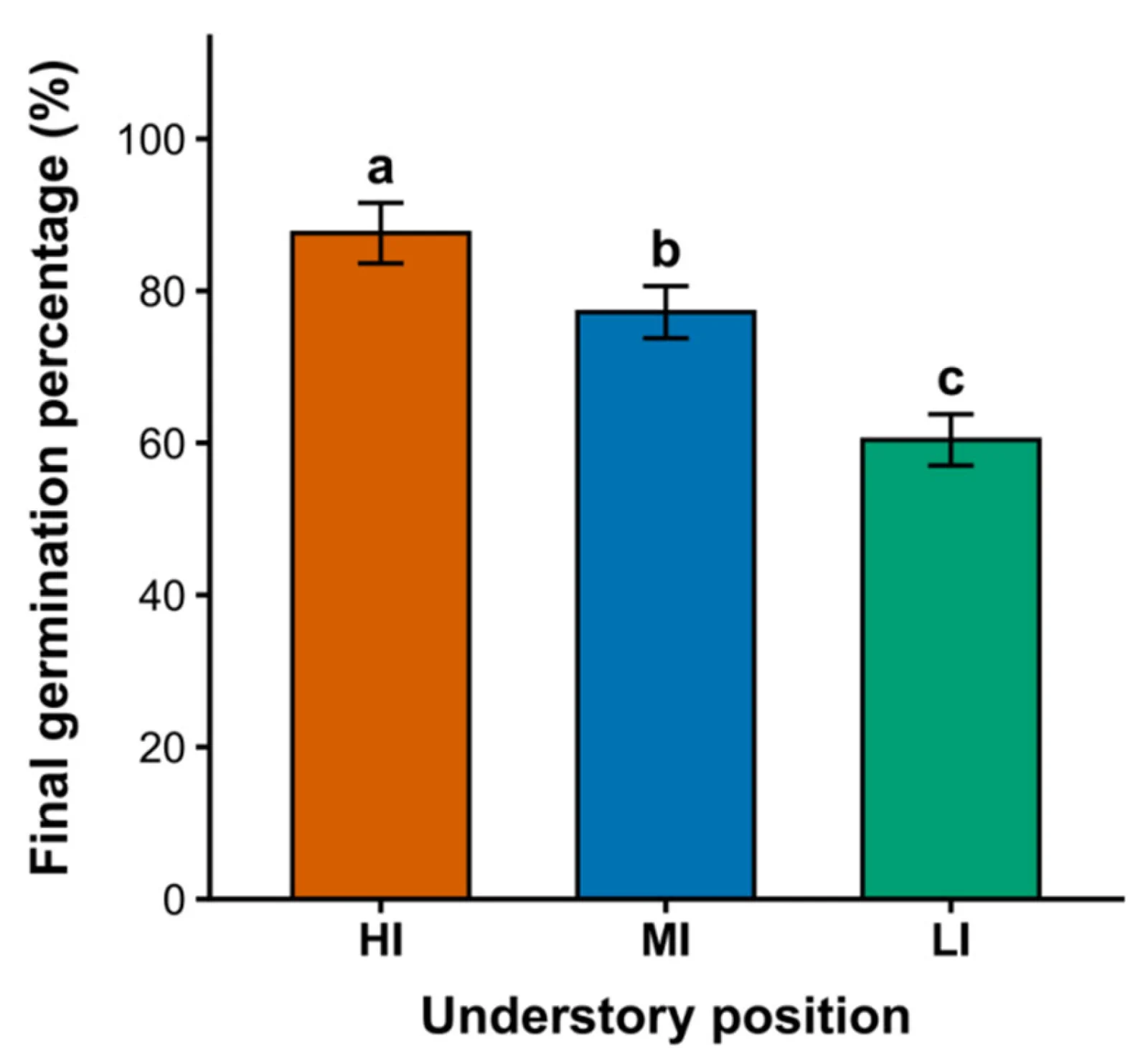
Final germination percentage of *P. ostii* seeds at three understory positions differing in light intensity. Values are means ± SD (*n* = 10). Different lowercase letters indicate significant differences at *p* < 0.05.

Final germination percentage differed significantly among the three positions (Figure 6; binomial GLM, *P* = 7.92 × 10^−23^). Seeds collected from plants at HI, MI, and LI had final germination percentages of 87.6%, 77.2%, and 60.4%, respectively, and all pairwise comparisons were significant (HI vs. MI, *P* = 6.01 × 10^−5^; HI vs. LI, *P* = 2.91 × 10^−14^; MI vs. LI, *P* = 4.08 × 10^−8^). The position × 100-seed weight interaction was not significant (*P* = 0.857). In the additive model, position remained significant (*P* = 3.78 × 10^−6^), whereas 100-seed weight was not significant (*P* = 0.430).

### 3.6. Integrated Trait Patterns and Correlations Across Understory Positions Differing in Light Intensity

**Figure 7:**
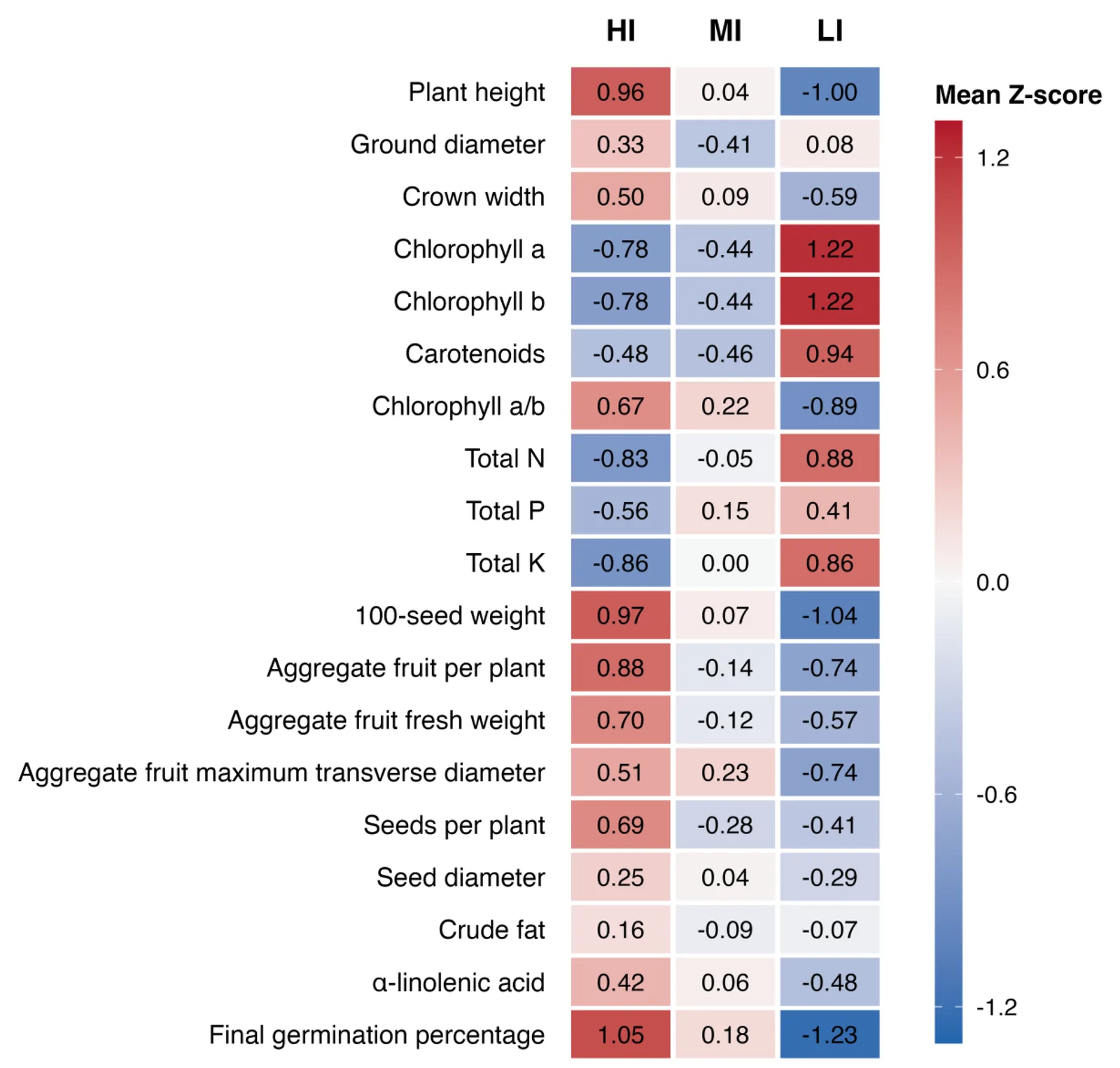
Standardized trait profiles of *P. ostii* across three understory positions. Values are mean Z-scores (*n* = 10).

The standardized heatmap revealed contrasting trait profiles between HI and LI, whereas MI generally showed intermediate standardized values (Figure 7). At HI, higher mean Z-scores were observed for plant height, crown width, 100-seed weight, aggregate fruit per plant, aggregate fruit fresh weight, aggregate fruit maximum transverse diameter, seeds per plant, α-linolenic acid, and final germination percentage. In contrast, LI was characterized by higher standardized values for chlorophyll a, chlorophyll b, carotenoids, total N, total P, and total K, but lower values for most growth, aggregate fruit, seed, and germination-related traits. These standardized values indicate the direction of the position-level trait profiles and do not themselves represent statistically significant differences.

**Figure 8:**
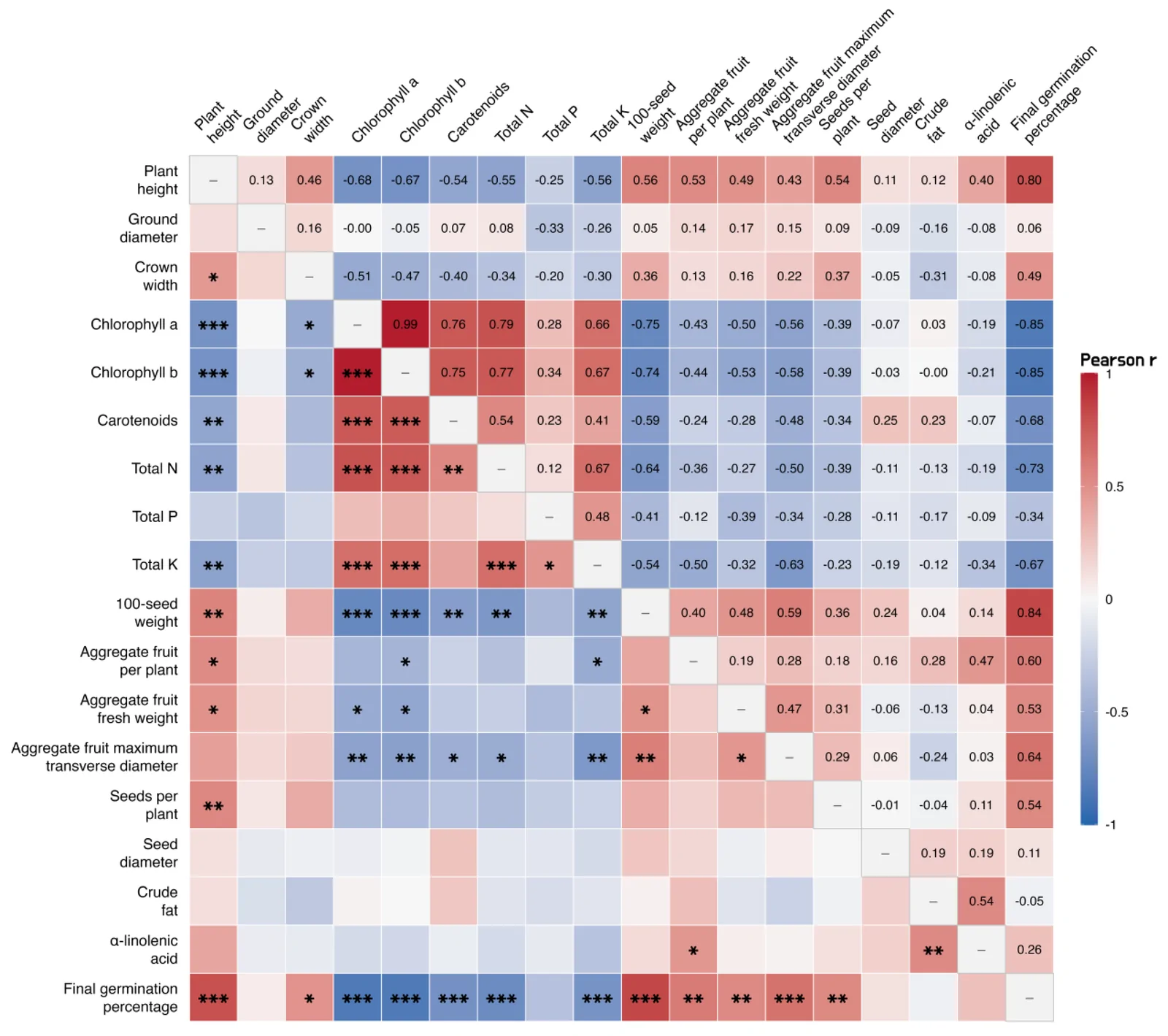
Pearson correlations among measured traits across all 30 sampled plants pooled from the three understory positions. Asterisks indicate significance after Benjamini–Hochberg FDR correction (* q < 0.05, ** q < 0.01, *** q < 0.001).

In the pooled analysis across all 30 plants, final germination percentage was positively correlated with 100-seed weight (r = 0.838, FDR-adjusted *P* = 2.88 × 10^−7^), plant height (r = 0.799, FDR-adjusted *P* = 3.64 × 10^−6^), crown width, aggregate fruit traits, and seeds per plant (Figure 8). Final germination percentage was negatively correlated with chlorophyll a (r = −0.852, FDR-adjusted *P* = 1.79 × 10^−7^), chlorophyll b, carotenoids, total N, and total K. However, after removing position effects, these correlations with final germination were no longer significant after FDR correction. For example, the correlation between germination and 100-seed weight decreased to r = 0.257 (FDR-adjusted *P* = 0.652), whereas that between germination and chlorophyll a decreased to r = −0.186 (FDR-adjusted *P* = 0.673). Thus, the strong correlations observed in the pooled analysis largely reflected differences among the three sampled positions rather than within-position trait covariation.

## 4. Discussion

### 4.1. Microclimatic Variation Among Understory Positions Differing in Light Intensity

The HI, MI, and LI positions beneath the pecan canopy differed markedly in understory light intensity. PAR consistently followed the order HI > MI > LI in both summer and late autumn, with greater differences among the three positions in summer. This pattern reflects spatial variation in pecan canopy openness and the resulting distribution of solar radiation within the agroforestry system. Similar spatial heterogeneity is common in tree–crop systems, where understory light intensity varies with canopy structure, position relative to tree rows, and solar elevation [25]. The three positions therefore represented distinct levels of understory light intensity within the same pecan-based agroforestry system.

Air temperature and relative humidity varied less consistently than PAR and were more dependent on season and time of day. In summer, air temperature tended to be higher at HI, whereas relative humidity was higher at LI at several measurement times. These patterns suggest that greater canopy cover moderated daytime microclimatic conditions by reducing incoming radiation. Comparable effects of tree canopies on understory temperature and humidity have been reported in other agroforestry systems [26]. In late autumn, however, differences in air temperature and relative humidity among the three positions were relatively small. Thus, canopy-related modification of the understory microclimate was more evident under the higher radiation conditions of summer. Light conditions beneath the pecan canopy were characterized using PAR measurements. Because spectral composition, UV radiation, and red:far-red and red:blue ratios were not measured, the observed responses should be interpreted as associated with differences among the three sampling positions rather than with PAR intensity alone.

### 4.2. Growth Responses Across Understory Positions Differing in Light Intensity

Plant height showed a decreasing pattern from HI to LI, but was significantly lower only at LI than at HI and MI. Crown width was lower at LI than at HI, whereas MI was intermediate, and ground diameter did not differ significantly among the three positions. Unlike plant height and crown width, ground diameter did not follow the PAR gradient, with mean values following the order HI > LI > MI. This non-monotonic pattern may reflect pre-existing plant-to-plant or position-related variation. Because baseline measurements of plant size were not available, the observed ground-diameter pattern cannot be attributed specifically to differences in understory light intensity.

The lower plant height at LI and the greater crown width at HI than at LI were broadly consistent with differences in PAR availability among the sampled positions, although the observational design does not allow these differences to be attributed to PAR alone. Notably, plant height was lower at LI rather than showing the elongation commonly associated with shade-avoidance responses. This pattern is consistent with the possibility that, under the more heavily shaded conditions, limitations on carbon acquisition outweighed any elongation response, although this interpretation remains tentative because spectral quality and gas exchange were not measured. Reduced PAR limits photosynthetic carbon gain and the assimilates available for shoot and canopy development, particularly when shading persists during active growth [27]. However, gas-exchange parameters were not measured in the present study; therefore, differences in photosynthetic carbon gain among the three positions were not directly demonstrated, and this mechanism should be regarded as an inference based on previous studies. Comparable responses have been reported in other woody species. In Douglas-fir (*Pseudotsuga menziesii*) saplings, height growth and crown development increase with increasing light availability [28], whereas white meadowsweet (*Spiraea alba*) performs better under full light or moderate shade than under deep shade [29]. These findings are consistent with the lower plant height and crown width observed at LI. However, because PAR co-varied with other unmeasured environmental factors among the three positions, the observed growth differences cannot be attributed to light availability alone.

### 4.3. Leaf Photosynthetic Pigment and Mineral Nutrient Responses Across Understory Positions Differing in Light Intensity

The concentrations of chlorophyll a, chlorophyll b, and carotenoids were significantly higher at LI than at HI and MI. Increased pigment concentrations are a common leaf response to reduced light and may improve light capture under low irradiance [17]. Similar responses have been reported in rice (*Oryza sativa*) [30] and tea plant (*Camellia sinensis*) [31].

Consistent with the pigment pattern, the chlorophyll a/b ratio was lower at LI than at HI and MI, with mean values of 3.15, 3.06, and 2.83 at HI, MI, and LI, respectively. This decline reflects a proportionally greater increase in chlorophyll b at the lowest-light position and is consistent with adjustment of the light-harvesting system under lower irradiance.

Carotenoids also contribute to energy transfer and photoprotection, helping to maintain the stability of the photosynthetic apparatus [32]. The coordinated increases in chlorophylls and carotenoids at LI were therefore consistent with leaf-level acclimation to lower light intensity. However, because SLA or LMA was not measured, the mass-based pigment data alone cannot distinguish physiological acclimation from differences associated with leaf structure.

Leaf N, P, and K concentrations were generally higher at the more shaded positions. N is a major component of chlorophyll, Rubisco, and other photosynthetic proteins; and K is involved in stomatal regulation, osmotic adjustment, enzyme activation, and assimilate transport [33,34]. Similar results have also been reported in other plants. For example, white clover (*Trifolium repens*) seedlings showed higher leaf nitrogen content per unit dry mass under lower irradiance [35], and bell pepper (*Capsicum annuum*) showed increased concentrations of leaf N, K, Ca, and Mg with increasing shading level [36]. However, because these nutrient concentrations were expressed on a leaf dry-mass basis, the higher values at LI do not necessarily indicate greater nutrient uptake or whole-plant nutrient accumulation. They may partly reflect slower biomass production, altered nutrient allocation, or a weaker dilution effect under low-light conditions.

### 4.4. Seed Yield Responses Across Understory Positions Differing in Light Intensity

Plants at HI had greater 100-seed weight, aggregate fruit per plant, aggregate fruit fresh weight, seeds per plant, and aggregate fruit maximum transverse diameter than plants at LI, whereas seed diameter was similar among the three positions. This pattern suggests that variation in understory light intensity was more strongly associated with aggregate fruit formation, aggregate fruit development, seed number, and seed mass than with external seed size. Reproductive development depends on continued carbon assimilation and the transport of assimilates from source leaves to developing aggregate fruits and seeds [37,38]. However, because flower number and fruit set were not recorded, the higher aggregate fruit number at HI cannot be attributed specifically to differences in assimilate supply. Differences in pollinator activity and fruit set among the sampled positions represent plausible alternative explanations, as pollination has been shown to affect fruit set and seed yield in *P. ostii* [13,14].

In the present study, 100-seed weight was determined using fresh seeds immediately after harvest. However, seed moisture content at weighing was not measured, and the fresh-weight basis alone cannot fully explain the difference between the present values and previously reported fresh-seed values. The relatively high 100-seed weight observed here may also reflect differences in plant material, developmental stage at harvest, environmental conditions, and field management. In addition, flowering occurred later at MI and LI, whereas all three positions were harvested on the same calendar date. Consequently, seeds from the more heavily shaded positions had a shorter developmental period before harvest, and differences in developmental age at harvest may also have contributed to the lower 100-seed weight observed at LI.

Similar responses have been reported in other oilseed crops. In sacha inchi (*Plukenetia volubilis*), shading delayed flowering and reduced photosynthesis, growth, and yield [39]. The lower aggregate fruits and seed traits observed at LI are broadly consistent with these findings. The similarity in seed diameter among the three positions, despite clear differences in 100-seed weight and seeds per plant, suggests that light intensity was more strongly related to seed filling and reproductive output than to external seed dimensions. Previous research on *P. ostii* has shown that reserve accumulation during seed maturation is closely linked to seed development and oil formation [40]. Overall, aggregate fruit per plant, seeds per plant, and 100-seed weight were more responsive to differences in light intensity among positions than seed diameter. Relatively open positions beneath the pecan canopy were therefore more favorable for aggregate fruit development and seed filling, whereas persistently low light intensity was associated with poorer reproductive performance.

### 4.5. Seed Oil Quality and Germination Responses Across Understory Positions Differing in Light Intensity

Crude fat content and α-linolenic acid content did not differ significantly among the three positions, although the absolute α-linolenic acid contents were lower than previously reported values for *P. ostii* kernels [4,6]. In other woody oilseed crops, including olive (*Olea europaea*), fruit light exposure and canopy position can influence fatty acid composition and oil development [41,42].

Although mean α-linolenic acid content decreased numerically from HI to LI, the differences among positions were not statistically significant. Therefore, the present data do not provide strong evidence that α-linolenic acid content differed among the sampled understory positions. Because temperature, light availability, and seed developmental status co-varied spatially, their individual contributions to fatty acid variation cannot be separated in the present observational design.

Final germination percentage differed significantly among the three sampled positions and followed the order HI > MI > LI. Seed germination in *P. ostii* is strongly influenced by dormancy-release conditions and the physiological status of the seeds [43,44]. In the pooled dataset, final germination percentage was positively correlated with 100-seed weight. However, the binomial model including both position and 100-seed weight showed that the position effect remained significant, whereas 100-seed weight was not significant. This indicates that differences in seed mass alone did not explain the lower germination percentage observed at LI. The persistence of a significant position effect after accounting for 100-seed weight is also consistent with the influence of a position-level factor not captured by seed mass. One such factor is developmental age at harvest, because flowering was delayed at MI and LI while all positions were harvested on the same calendar date. Thus, the lower germination percentage at LI may partly reflect differences in seed developmental status at harvest, although the present design cannot separate this effect from other position-associated environmental factors. Within the sampled orchard, final germination was highest at HI and lowest at LI, but the underlying causes cannot be attributed to seed mass or light availability alone.

### 4.6. Integrated Trait Responses Across Understory Positions Differing in Light Intensity

The standardized heatmap revealed a clear contrast between leaf-level responses and whole-plant performance. LI was characterized by higher photosynthetic pigment and mineral nutrient concentrations, with the pigment pattern being consistent with leaf-level responses to lower light intensity. However, plant height, crown width, seed yield components, and final germination percentage were lower at LI. This pattern is consistent with the general view that phenotypic plasticity has ecological limits and may not fully compensate for strong environmental constraints [45,46]. In the present study, the higher leaf pigment and mineral nutrient concentrations observed at LI were not accompanied by better vegetative growth, reproductive performance, or seed germination.

Previous studies have shown that moderate shading alleviates summer high-temperature and high-light stress and improves leaf photosynthetic performance in *P. ostii.* However, the light range that best balances leaf physiological performance and reproductive output remains unclear across studies. Previous results have indicated that exposure to approximately 80% of full sunlight improves microclimatic conditions, mitigates heat damage, and enhances photosynthetic capacity, whereas stronger shading reduces seed yield and oil quality [18].

Among the three sampled positions, HI showed the highest values for several seed yield components and final germination percentage. At 13:00 on 14 July, HI, MI, and LI received 40.3%, 17.3%, and 6.6% of simultaneous open-sky PAR, respectively. On the full-sun scale used in ref. [18], even HI was closest to the moderate-shading level, whereas MI and LI were at or below the severe-shading level. Thus, none of the positions sampled in the present study represented the relatively high-light range in which improved seed yield and oil quality were reported in ref. [18]. This difference in the sampled light range helps reconcile the present observations with that previous study. Differences among studies may also reflect variation in climatic conditions, genetic background, field management, and plant developmental stage. Taken together, the present results are broadly consistent with previous studies showing that excessive shading can constrain the performance of *P. ostii*. However, the present observational design does not allow an optimal light range to be defined. Because crop species differ markedly in their responses to shade, the balance between canopy-mediated microclimatic moderation and understory light availability requires further evaluation [47].

Several limitations of the observational design should be considered when interpreting the present results. HI, MI, and LI represented naturally occurring positions within a single orchard rather than independently replicated light treatments. Consequently, understory PAR may have co-varied with other spatial factors, including soil moisture, soil chemical properties, below-ground competition, root distribution, and pre-existing plant size. These variables, as well as direct measurements of canopy openness or LAI, were not collected during the original field survey and therefore cannot be reconstructed retrospectively. Baseline measurements of plant size were also unavailable. In addition, plants were selected non-randomly based on the criterion of being visibly healthy and free of symptoms of pests or diseases, which may have underrepresented plants in poorer condition, and therefore limits the generalization of the results to all plants within each understory position. Accordingly, the observed trait differences should be interpreted as associations with the sampled understory positions and their corresponding PAR environments rather than as responses caused by PAR alone. Future studies should use independently replicated canopy-management treatments and incorporate measurements of soil conditions and below-ground competition to separate the effects of light from other spatially covarying factors. Future experiments should also harvest seeds at a comparable physiological maturity rather than on a common calendar date. In addition, microenvironmental measurements were conducted on only two dates and therefore do not characterize understory light conditions throughout the entire April–July reproductive and seed-filling period.

## 5. Conclusions

Under the conditions of this study, the sampled understory positions differed in growth, reproductive traits, and final seed germination of *P. ostii*. Plants at LI had higher leaf photosynthetic pigment and mineral nutrient concentrations, whereas plant height, several seed-yield traits, and final germination percentage were lower than at the less-shaded positions. Crude fat and α-linolenic acid contents did not differ significantly among the three positions. Overall, HI was characterized by higher values for several growth and reproductive traits and by the highest final germination percentage.

Within the sampled orchard, relatively open positions with higher PAR were associated with better vegetative growth, reproductive performance, and seed germination than more heavily shaded positions. These observations suggest that the balance between canopy-mediated microclimatic moderation and understory light availability warrants further evaluation in replicated canopy-management experiments. Further studies combining multi-year observations with replicated canopy-management treatments are needed to determine the range of understory light intensity suitable for *P. ostii* production in pecan-based agroforestry systems.

## Data Availability

The plant-level data supporting the findings of this study are available from the corresponding author upon reasonable request.
